# Acute Hemiparesis in a Healthy Elderly Woman: Where and What Is the Lesion?

**DOI:** 10.3389/fneur.2017.00109

**Published:** 2017-03-21

**Authors:** Ji Hoon Lee, Sung Hyuk Heo, Jin San Lee, Dae-Il Chang, Ki-Ho Park, Ji-Youn Sung, Il Ki Hong, Myeong Hee Kim, Bong Jin Park, Woo Suk Choi

**Affiliations:** ^1^Department of Neurology, Kyung Hee University College of Medicine, Seoul, South Korea; ^2^Department of Internal Medicine, Kyung Hee University College of Medicine, Seoul, South Korea; ^3^Department of Pathology, Kyung Hee University College of Medicine, Seoul, South Korea; ^4^Department of Nuclear Medicine, Kyung Hee University College of Medicine, Seoul, South Korea; ^5^Department of Laboratory Medicine, Kyung Hee University College of Medicine, Seoul, South Korea; ^6^Department of Neurosurgery, Kyung Hee University College of Medicine, Seoul, South Korea; ^7^Department of Radiology, Kyung Hee University College of Medicine, Seoul, South Korea

**Keywords:** cerebritis, myelitis, hemiparesis, *Propionibacterium acnes*, clinical pathology

## Abstract

Hemiparesis may be the result of lesions in the contralateral pyramidal tract in the brain or, less frequently, in the ipsilateral pyramidal tract in the upper cervical spinal cord. However, although rare, multiple lesions that simultaneously occur in both of these regions may be the cause of acute hemiparesis, and the clinical symptoms can often be misdiagnosed as a stroke. In addition, the correct diagnosis of these multiple central nervous system (CNS) lesions is very challenging if they are caused by infection from an unexpected microorganism. We evaluated an elderly healthy woman who presented with acute hemiparesis and multiple brain and spinal cord lesions that were confirmed to occur from an infection with *Propionibacterium acnes*. In this report, the differential diagnosis and histopathological findings are discussed for these multiple CNS lesions in this healthy woman.

## Case Presentation

A 75-year-old healthy woman presented at our department with weakness in her left side that had suddenly developed during the previous few days. She did not complain of fever, headache, and nausea or vomiting. She did not have a history of significant medical issues such as hypertension, diabetes, and heart disease or a recent history of travel, head trauma, or drug consumption. She had received tattoos on her eyebrows several decades prior, but there was no clinical history of skin rash including aphthous or genital ulceration. Her mentality was alert, and cognition was normal, but the initial neurological exam revealed left facial palsy with intact forehead activation and distally predominant left hemiparesis. Motor power was grade 4 on the Medical Research Council (MRC) scale in the left proximal upper extremities such as deltoid, biceps brachii, and triceps brachii, grade 3 in the left distal upper extremities such as wrist extension, wrist flexion, finger extension, and finger abduction, and grade 4 in the left lower extremities including iliopsoas, quadriceps, hamstrings, tibialis anterior, gastrocnemius, extensor halluces longus, and flexor halluces longus. There were no sensory symptoms or signs. Deep tendon reflexes were brisk in the left extremities with extensor plantar responses. Meningeal irritation signs were absent.

**Figure 1:**
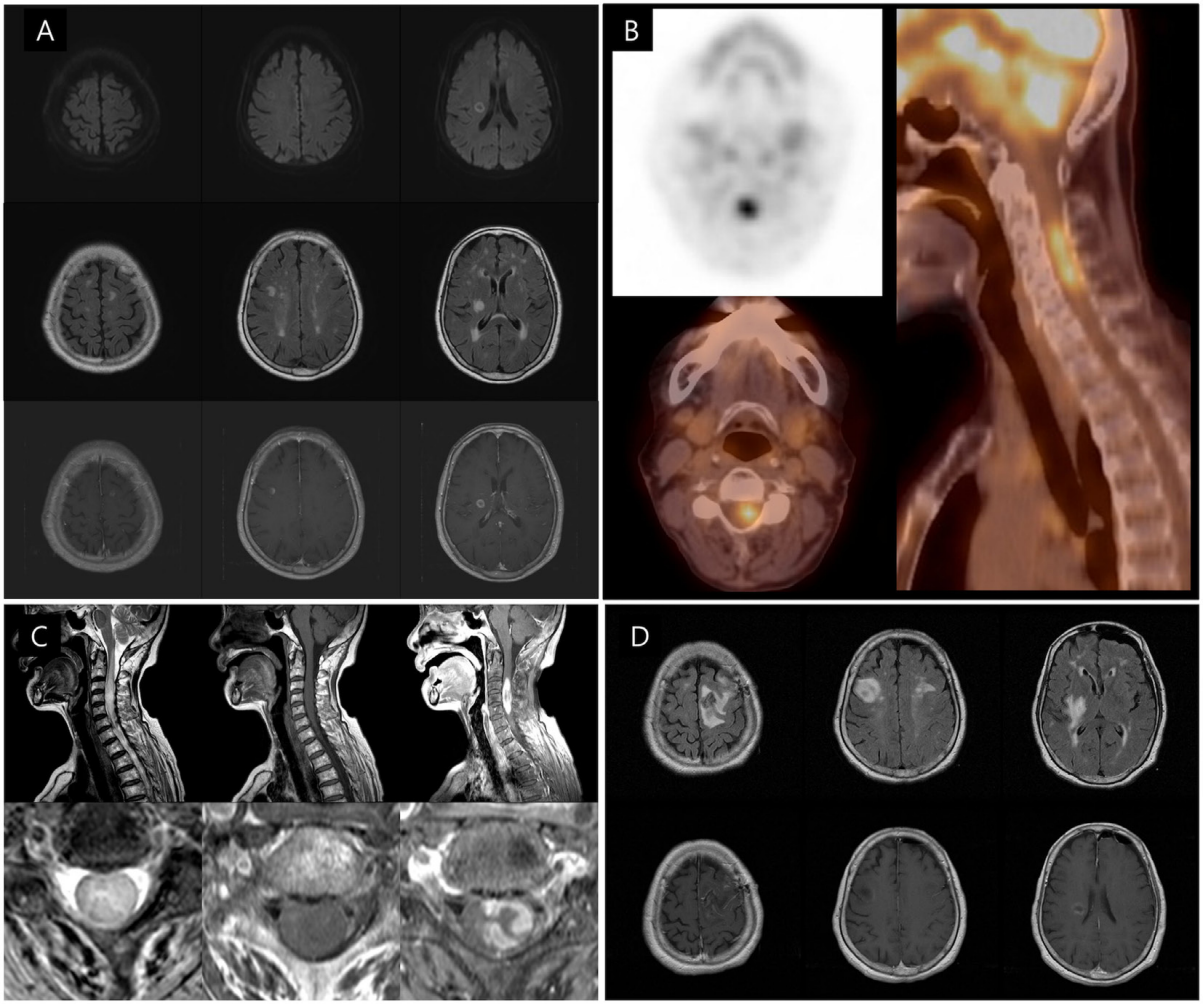
**Initial and follow-up brain and spinal cord MRI and F-18 FDG PET/computed tomography (CT) scans of the patient**. **(A)** Diffusion-weighted image (upper row) exhibiting small, round, high, and central low-signal-intensity lesions in the right frontal lobe and internal capsule. T2 fluid attenuation inversion recovery (FLAIR) image (middle row) showing multiple small high-signal-intensity lesions in both cerebral hemispheres. Enhanced T1-weighted image (lower row) demonstrating rim enhancement in both the frontal cortical and internal capsular lesions. **(B)** F-18 FDG PET/CT scan showing another high-uptake lesion in the patient’s neck. **(C)** The cervical spinal T2 FLAIR image revealing high-signal-intensity lesions with surrounding edema at the C2–C6 level of the spinal cord, especially the left pyramidal tract. The cervical spinal enhanced T1-weighted image displaying heterogenous enhancement, at the same level. **(D)** The follow-up T2 FLAIR and enhanced T1-weighted images performed after biopsy showing that both frontal cortical and subcortical high-signal-intensity lesions have increased in size.

The results of blood work showed a normal complete blood cell count, electrolyte levels, liver function tests, and coagulation test. Initial erythrocyte sedimentation rate and C-reactive protein were also normal, but there were positive results for syphilis [serum rapid plasma regain (RPR) test and *Treponema pallidum* hemagglutination assay]. Electrocardiogram and echocardiogram were normal. An initial brain magnetic resonance imaging (MRI) scan revealed multiple small lesions in both the frontal cortex and subcortex that primarily involved the right internal capsule. In addition, rim enhancement was observed on the gadolinium-enhanced T1 images (Figure 1A). There were no abnormal findings in intra- and extra-cranial MR angiography (Figure S1 in Supplementary Material). The initial clinical impression was the presence of multiple metastatic brain tumors or infections such as neurosyphilis and bacterial abscesses, so additional diagnostic workups were performed sequentially. Serologic tests for antinuclear antibody, antineutrophil cytoplasmic antibody, and human immunodeficiency virus (HIV) were negative, and rheumatoid factor and complement 3 and 4 level (C3 and C4) were within normal range. Results of serologic test for tumor marker were normal in CA15-3, CA 19-9, CEA, and AFP level, but CA 125 level was elevated approximately above 10 times above normal range (392.2 U/mL).

Two days after her admission, the patient’s left hemiparesis was worsened (MRC grade 1 in the left upper and lower limbs including deltoid, biceps brachii, triceps brachii, wrist extension, wrist flexion, finger extension, finger abduction, iliopsoas, quadriceps, hamstrings, tibialis anterior, gastrocnemius, extensor halluces longus, and flexor halluces longus). There were no other neurological changes such as sensory loss, and additional laboratory findings including blood culture and procalcitonin levels were normal. Results of cerebrospinal fluid (CSF) analysis demonstrated normal level of protein (41.4 mg/dL), glucose (58.4 mg/dL), and cell count (white blood cell: 1/mm^3^ and red blood cell: 0/mm^3^), and a CSF fluorescent treponemal antibody absorption (FTA-ABS) IgG analysis showed minimal reactivity. An FTA-ABS IgM analysis was non-reactive, and a CSF Venereal Disease Research Laboratory test, CSF cytology, and cultures for mycobacterium and fungi were negative. Her initial oncologic workups, including abdomen and chest computed tomography (CT) scans, did not show any lesions that suggested primary cancer. After consultation with oncologist, an F-18 fluorodeoxyglucose positron emission tomography (^18^F-FDG PET)-CT scan, which was subsequently performed to determine the presence of any hidden malignancies revealed a hypermetabolic lesion in the cervical spinal cord (Figure 1B). A cervical MRI scan revealed high signal intensities along the C2–C6 level of the spinal cord with heterogeneous enhancement, including the left pyramidal tract (Figure 1C). To identify the etiology of the multiple central nervous system (CNS) lesions, a brain biopsy of the left frontal lobe lesion was performed. A follow-up MRI scan taken just after the brain biopsy revealed additional increases in sizes and numbers of the multiple brain lesions (Figure 1D).

**Figure 2:**
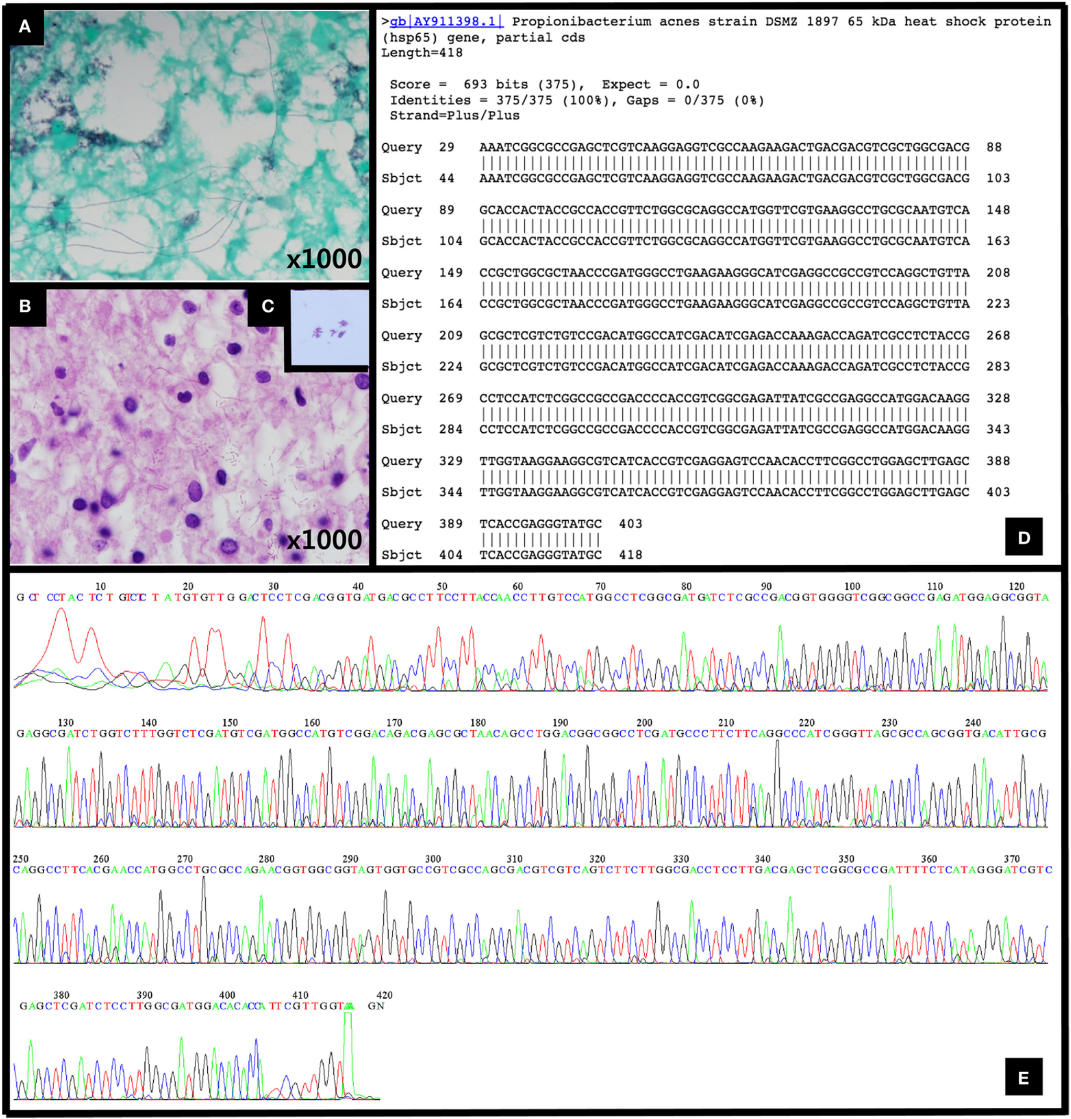
**(A)** Gomori methenamine silver stain (1,000×), **(B)** hematoxylin and eosin (H&E) stain (1,000×), and **(C)** Gram stain (×1,000) revealing Gram-positive bacilli. **(D,E)** The DNA sequencing results for the 65 kDa heat shock protein (Hsp65) gene of *Propionibacterium acnes* strain were entirely consistent with a *P. acnes* infection.

A histopathological examination revealed the localized collection of foamy histiocytes with mild perivascular lymphocytic infiltration in the brain tissue. Additionally, an unknown type of microorganism with a filamentous branching rod shape was found within the lesion, namely, in the cytoplasm of the foamy histiocytes, fibrillary brain tissue, and perivascular spaces. The microorganism was Gram-positive, had a positive Gomori methenamine silver test, a negative acid-fast bacteria test, and a negative Warthin–Starry test; these findings indicate that it was a Gram-positive microorganism, but its specific identification could not be determined (Figures 2A–C). Empirical antibiotic therapy that included sulfamethoxazole/trimethoprim with amikacin was initiated to cover the treatment of the most likely pathogenic organisms, such as *Nocardia* and *Actinomyces*. At the same time, DNA sequencing was requested for a confirmatory diagnosis. The patient showed elevated levels of liver enzymes 1 week after treatment, and the sulfamethoxazole/trimethoprim therapy was accordingly changed to imipenem. Approximately 1 month after the empirical treatment was initiated, the patient exhibited symptomatic improvements. The results of the DNA sequencing were entirely consistent with an infection with *Propionibacterium acnes* (Figures 2D,E).

**Figure 3:**
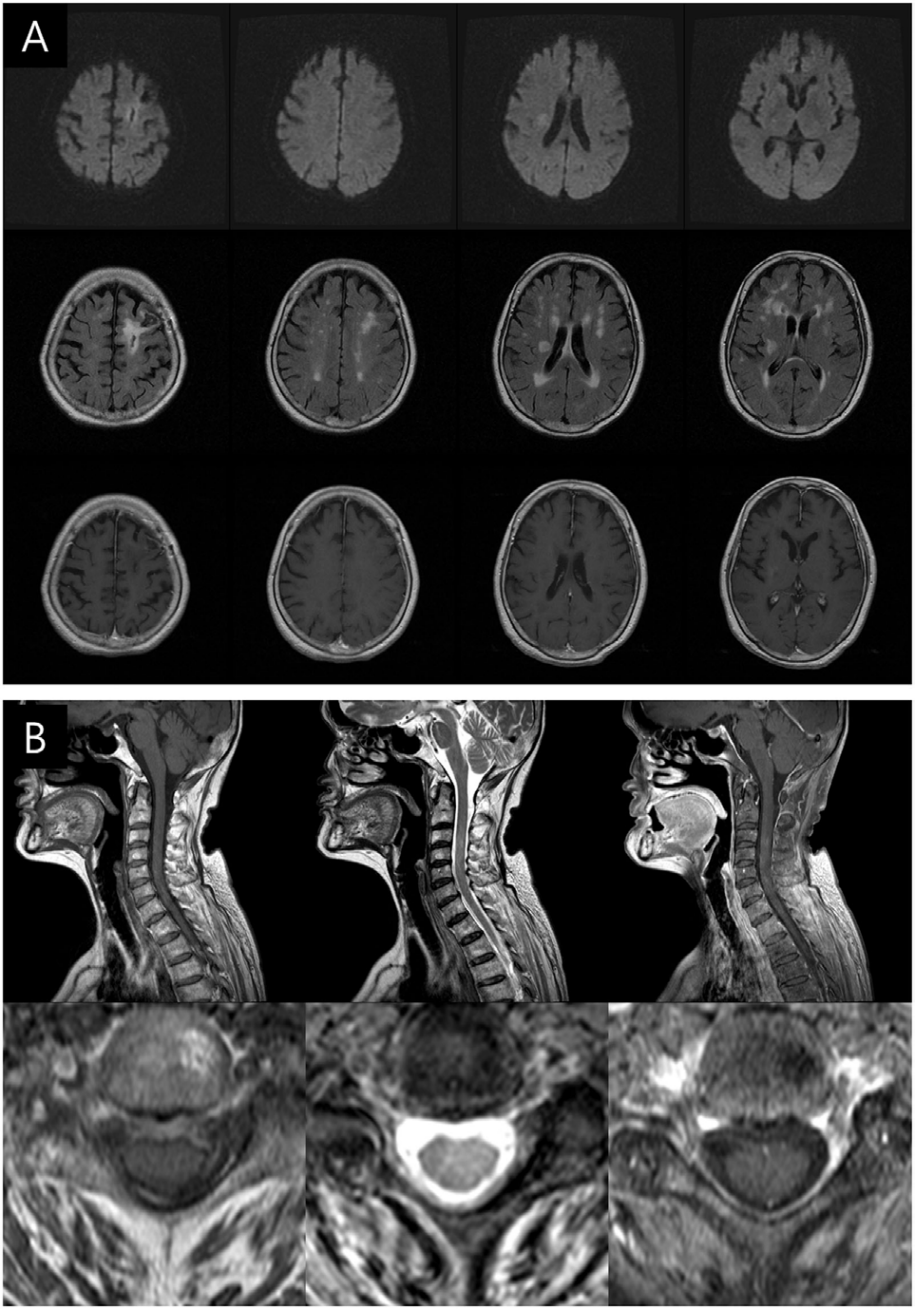
**Follow-up brain and cervical spine magnetic resonance imaging (MRI) scans taken 3 months after the maintenance of antibiotic treatment**. **(A)** Diffusion-weighted imaging (upper row), T2 fluid attenuation inversion recovery (middle row), and enhanced T1-weighted axial images (lower row) showing marked decrease in size of multiple lesions and perilesional edema. **(B)** Cervical spine MRI (T1, T2, and enhanced T1) displaying the nearly disappeared of high-intensity lesions in the cervical spinal cord.

Considering the sensitivity of *P. acnes* to imipenem and the improvement in the patient’s status, antibiotic therapy was continued. Follow-up brain and cervical spine MRI scans taken 3 months after the initiation of the antibiotic maintenance therapy showed nearly resolved lesions (Figure 3) concomitant with amelioration of her left hemiparesis (MRC grades of 4 or above). After 6 months, she was able to sit and eat alone, and any neurological sequelae except weakness were not remained.

## Discussion

The present report describes a case of acute hemiparesis in a healthy 75-year-old female. Two important points were demonstrated by this report. First, the two distinct lesions causing the hemiparesis of the patient (in the contralateral pyramidal tract in the brain and the ipsilateral pyramidal tract in the cervical spinal cord) were discovered at the same time using standard techniques, but the subsequent exacerbation of the patient’s initial symptoms required the use of additional sequential modalities to make a differential diagnosis. Second, the etiology of the multiple CNS lesions was determined to be a CNS infection caused by *P. acnes*; this was confirmed by a brain biopsy and DNA sequencing. This type of CNS infection is very rare in healthy adults.

Hemiparesis may be caused by any type of lesion that results in damage within the system that extends from the primary motor cortex to the intramedullary pyramidal tract in the upper cervical spinal cord. However, it is difficult to determine whether cervical spinal lesions are present in elderly patients with intracranial lesions, especially when they are concomitant with cranial neuropathies such as dysarthria or hemi facial palsy. Previous reports have described acute cervical spinal cord lesions that were misdiagnosed as acute ischemic strokes (1, 2). In addition to her hemiparesis, the patient in the current study also presented with central-type facial palsy, so it was strongly suggested that the underlying pathology was an intracranial lesion. However, small internal capsule lesion of the patient was not enough to explain a rapid progression of weakness, and negative CSF findings and an elevated tumor marker warranted a subsequent oncologic workup. After consultation with an oncologist, the ^18^F-FDG PET-CT scan was performed to identify any hidden malignancies and to discover other CNS lesions. In these respects, it is suggested that clinicians exercise constant vigilance concerning the possible existence of extracerebral lesions in patients with hemiparesis and that assertive workups are necessary to make a differential diagnosis.

The differential diagnosis for multiple CNS lesions in an otherwise healthy elderly woman is exceedingly broad. It includes neoplasm (primary or metastatic); demyelinating diseases such as multiple sclerosis (MS), acute disseminated encephalomyelitis, or neuromyelitis optica (NMO); granulomatous diseases such as sarcoidosis, Wegener’s granulomatosis, or neuro-Behcet; and infectious diseases such as neurosyphilis, tuberculosis, other bacterial abscesses, or fungal infections.

Regarding the patient’s age, the possibility of multiple metastatic CNS tumors and the existence of underlying primary hidden malignancy needed to be excluded first. However, the abdominal and chest CT scans performed for screening about hidden malignancies, and the ^18^F-FDG PET-CT scan did not show any specific abnormal lesions suggesting malignancy. In addition, the result of CSF cytology was also negative. Although the patient had no history of demyelinating disease, the multiple brain and intramedullary cervical cord lesions can be considered as the first presentation of CNS demyelinating disease such as MS or NMO. However, normal results of the CSF study without albuminocytologic dissociation, rim enhancement of brain lesions in the T1-enhanced MRI, and the lack of optic neuritis indicated against these diagnoses. Because of normal immunologic tests and no clinical history of skin rash, the possibilities for autoimmune diseases, such as systemic lupus erythematosus, Sjogren syndrome, and neuro-Behcet disease, or granulomatous diseases, such as sarcoidosis and Wegener’s granulomatosis, were considered to be low.

Lumbar puncture is rarely warranted or sometimes contraindicated for brain abscess because of the significant risk such as herniation, especially in patients with focal neurologic symptoms or signs. However, because we found herniation risk of our patient was low in consideration of her brain image and clinical features, we performed CSF study to find the evidence of infection and rule out other disease processes including CNS vasculitis, demyelinating disease, and meningeal malignancy. The CSF pattern in the patients with brain abscess may reveal pleocytosis, elevated protein, and decreased glucose but will be infrequently positive (0–43%) in individuals (3–5). Consequently, although the CSF stain revealed no microorganisms and results of the CSF culture did not show any positive findings for bacterial or fungal growth, we could not exclude brain abscess. Other tests for *Toxoplasma, Sparganum, Clonorchis sinensis*, and *Paragonimus westermani* were all negative. In addition, although the serologic test for syphilis showed a positive result, RPR and FTA-ABS tests of CSF were non-reactive. The patient did not have any history of major head trauma or neurosurgery and was not in an immunocompromised state. She had received tattoos on her eyebrows, but this procedure was done several decades prior and did not result in any communicable infections. This type of tattoo is frequently performed on middle-aged women in Korea for cosmetic purposes. For this reason, if these multiple CNS lesions were caused by a CNS infection such as an abscess, it might be caused by an organism that we frequently encounter.

One systematic review reported potential causative microorganisms of brain abscess were *Streptococcus* species (34%), *Staphylococcus* species (18%), and Gram-negative enteric bacteria such as *Proteus* spp., *Klebsiella* spp., *Escherichia coli*, and *Enterobacteriaceae* (15%) (6). Because of the uncertain etiology of these lesions, despite a thorough evaluation, a brain biopsy was undertaken. From the specimen obtained at her left frontal lesion, an unknown type of microorganism with a filamentous branching rod shape was found. Empirical antibiotic therapy was initiated to cover the treatment of the most likely pathogenic organisms, until the result of DNA sequencing was determined.

*Propionibacterium acnes* is a Gram-positive anaerobic bacillus that is a part of the normal flora of the skin, oral cavity, large intestine, conjunctiva, and external ear canal; it is better known by the term acne vulgaris. *P. acnes* is primarily recognized as an opportunistic pathogen that causes a range of postoperative and device-related infections, including infections of the joints, mouth, eye, and brain (7). CNS infections that are related to *P. acnes* are highly correlated with the performance of neurosurgical procedures (8), after which the first symptoms can manifest anywhere from 12 days to 10 years (9, 10). A *P. acnes* infection in the CNS without a history of neurosurgical procedures is very rare, and these types of infections are also associated with various predisposing factors, such as HIV infection (11). Therefore, the spontaneous CNS infection by *P. acnes* is thought to be very rare in healthy adults.

Unlike previous reports, the present patient did not have any precipitating conditions that could induce opportunistic infections, except for having received tattoos on her eyebrows several decades prior. In addition, considering the long interval between the tattoo procedure and the disease outbreak in conjunction with the weak possibility that a pathogen could invade the CNS from superficial skin, the likelihood of an association between the tattoo and the delayed CNS infection was very low. Likewise, according to a meta-analysis of 9,699 patients with brain abscess, *Propionibacterium* spp. is a very rare causative microorganism comprising 0.7% of all cultured bacteria (6).

Two factors make the early diagnosis of a *P. acnes*-related CNS infection difficult to make. First, the identification of *P. acnes* as the cause of a CNS infection is very challenging, because it is difficult to isolate the bacteria due to its slow growth and anaerobic requirements. Second, the growth of *P. acnes* is considered a contaminant by many clinicians; thus, it is often underappreciated, which could lead to a delay in diagnosis and treatment, or even a missed diagnosis. Therefore, DNA sequencing is essential to confirm a diagnosis of CNS infection due to *P. acnes*, particularly when conventional methods of bacterial culturing are unable to identify a specific pathogen.

Over the past few decades, high-dose intravenous penicillin therapy in combination with surgical drainage and the removal of foreign bodies have been considered the gold standard of treatment for *P. acnes* CNS infections (12, 13). Recently, however, novel antibiotics such as cefotaxime, vancomycin, quinupristin/dalfopristin, and a combination of vancomycin and doxycycline have become more effective therapeutic options (14, 15). In the present case, imipenem was the primary antibiotic used for treatment following a change from sulfamethoxazole/trimethoprim, which was initially aimed toward targeting *Nocardia* or *Actinomyces* but had to be discontinued due to hepatotoxicity. Because imipenem effectively treated the *P. acnes* infection, and the patient exhibited improvements in her course of clinical symptoms, its administration was continued after acquiring the results of the DNA sequencing analyses, and an accurate prognosis was finally made.

All-cause mortality in patients hospitalized with abscess varies from 5 to 32% (4). Our patient showed improvement and completely treated, but neurological sequelae remained due to poor prognostic factors such as old age, rapid progression of clinical course, and delayed diagnosis caused by a rare pathogen (4–6). Therefore, it appears that the early administration of empirical antibiotic regimens that can fully cover and treat uncommon pathogens is important while quickly and accurately making a confirmative diagnosis of the specific pathogen underlying the clinical symptoms.

## Summary

To the best of our knowledge, the present case report is the first to describe multiple locations of CNS infection due to *P. acnes* in a healthy elderly patient without any other risk factors. Thus, the study indicates that continued vigilance and intensive workups, which include modern techniques such as PET-CT or DNA sequencing, are important for the accurate localization of lesions and the correct pathological diagnosis regarding unexpected and underestimated pathogens.

## Ethics Statement

Kyung Hee University Hospital Institutional Review Board (IRB) exempted IRB approval because our single case report did not perform any prospective intervention. In addition, written informed consent from the patient was obtained.

## Author Contributions

JHL: data collection and writing the manuscript. SH: conception and design of the study, data collection, and writing the manuscript. JSL, IH, and MK: data collection and interpretation. K-HP and J-YS: data collection and interpretation, pathologic review. D-IC, BP, and WC: critical revision of the manuscript for important intellectual content.

## Conflict of Interest Statement

The authors declare that the research was conducted in the absence of any commercial or financial relationships that could be construed as a potential conflict of interest. The reviewer CA and handling Editor declared their shared affiliation, and the handling Editor states that the process nevertheless met the standards of a fair and objective review.

## References

[1] Shoamanesh A Romero JR Kase CS Spontaneous cervical spinal epidural hematoma mimicking acute stroke. Can J Neurol Sci (2014) 41:533–4. 10.1017/S0317167100018655 24878486

[2] Liou KC Chen LA Lin YJ. Cervical spinal epidural hematoma mimics acute ischemic stroke. Am J Emerg Med (2012) 30:1322.e1–3. 10.1016/j.ajem.2011.06.018 21839603

[3] Tattevin P Bruneel F Clair B Lellouche F de Broucker T Chevret S Bacterial brain abscesses: a retrospective study of 94 patients admitted to an intensive care unit (1980 to 1999). Am J Med (2003) 115:143–6. 10.1016/s0002-9343(03)00292-4 12893401

[4] Patel K Clifford DB. Bacterial brain abscess. Neurohospitalist (2014) 4:196–204. 10.1177/1941874414540684 25360205 PMC4212419

[5] Muzumdar D Jhawar S Goel A. Brain abscess: an overview. Int J Surg (2011) 9:136–44. 10.1016/j.ijsu.2010.11.005 21087684

[6] Brouwer MC Coutinho JM van de Beek D. Clinical characteristics and outcome of brain abscess: systematic review and meta-analysis. Neurology (2014) 82:806–13. 10.1212/WNL.0000000000000172 24477107

[7] Perry A Lambert P. *Propionibacterium acnes*: infection beyond the skin. Expert Rev Anti Infect Ther (2011) 9:1149–56. 10.1586/eri.11.137 22114965

[8] Ramos JM Esteban J Soriano F. Isolation of *Propionibacterium acnes* from central nervous system infections. Anaerobe (1995) 1:17–20. 10.1016/S1075-9964(95)80366-1 16887502

[9] Kranick SM Vinnard C Kolson DL. *Propionibacterium acnes* brain abscess appearing 10 years after neurosurgery. Arch Neurol (2009) 66:793–5. 10.1001/archneurol.2009.75 19506144

[10] Nisbet M Briggs S Ellis-Pegler R Thomas M Holland D. *Propionibacterium acnes*: an under-appreciated cause of post-neurosurgical infection. J Antimicrob Chemother (2007) 60:1097–103. 10.1093/jac/dkm351 17875606

[11] Lyons JL Scripko PD Mukerji SS Awosika O Shieh WJ Zaki S *Propionibacterium acnes* brain abscess in a patient with HIV-1 infection. J Neurovirol (2012) 18:148–50. 10.1007/s13365-012-0088-z 22403028

[12] Collignon PJ Munro R Sorrell TC *Propionibacterium acnes* infection in neurosurgical patients. Experience with high-dose penicillin therapy. Med J Aust (1986) 145:408–10. 3762478

[13] Senneville E Savage C Lamy O Fawaz A Bourez J Ajana F Failure of intravenous antibiotic therapy of multiple temporal brain abscesses due to *Propionibacterium acnes* requiring temporal lobectomy. J Infect (1997) 34:269–71. 10.1016/S0163-4453(97)94479-0 9200038

[14] Fincher ME Forsyth M Rahimi SY. Successful management of central nervous system infection due to *Propionibacterium acnes* with vancomycin and doxycycline. South Med J (2005) 98:118–21. 10.1097/01.SMJ.0000149392.11493.8C 15678647

[15] Mory F Fougnot S Rabaud C Schuhmacher H Lozniewski A. In vitro activities of cefotaxime, vancomycin, quinupristin/dalfopristin, linezolid and other antibiotics alone and in combination against *Propionibacterium acnes* isolates from central nervous system infections. J Antimicrob Chemother (2005) 55:265–8. 10.1093/jac/dkh521 15590714

