# Supplementary figures and images for: Acute Hemiparesis in a Healthy Elderly Woman: Where and What Is the Lesion?
Source: Front Neurol. 2017 Mar 21;8:109. doi: 10.3389/fneur.2017.00109 (PMC5359233; doi:10.3389/fneur.2017.00109)

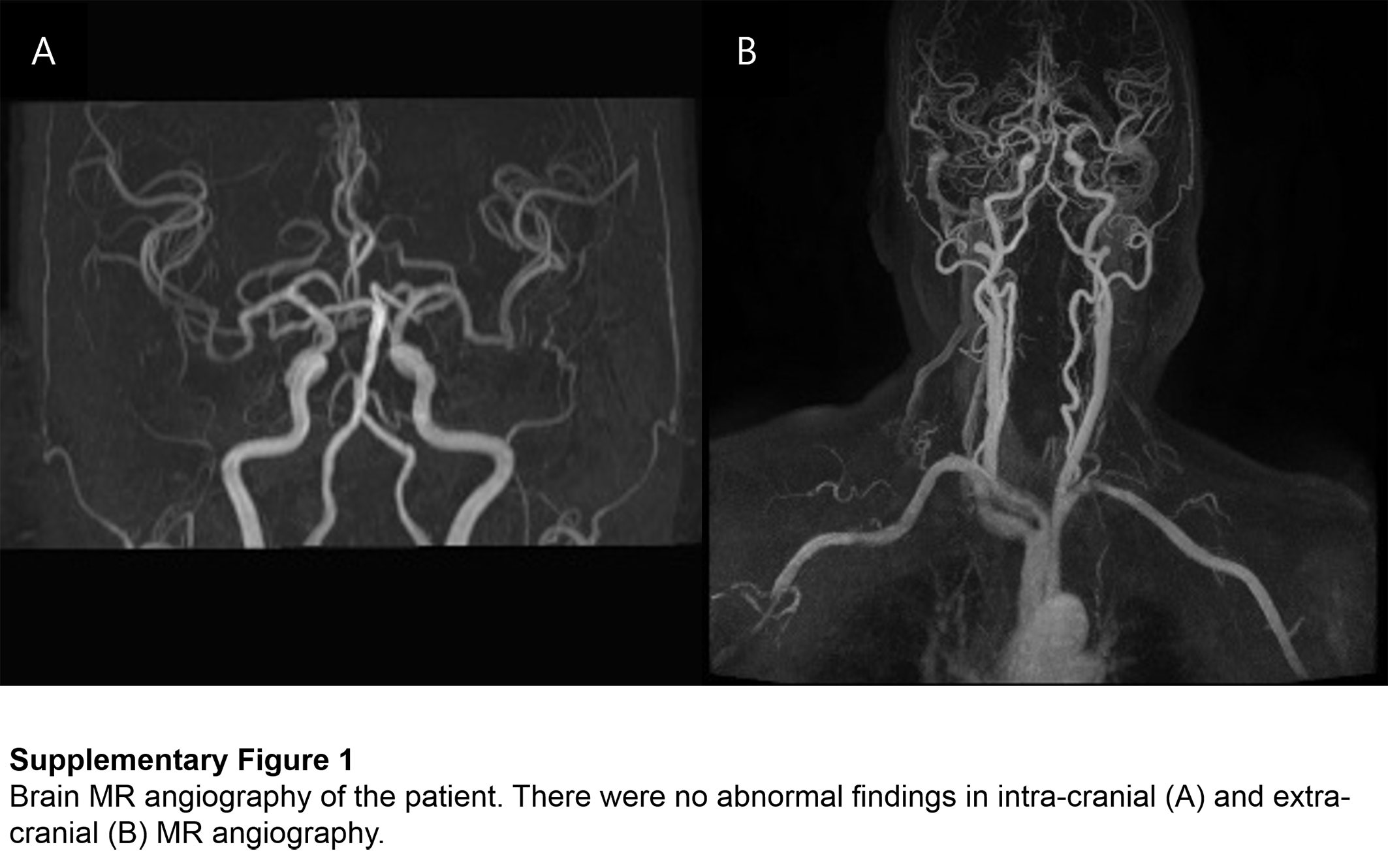

Supplement: Supplementary file 1 [file Image_1.tif]
